# The preventive effect of metformin on progression of benign prostate hyperplasia: A nationwide population-based cohort study in Korea

**DOI:** 10.1371/journal.pone.0219394

**Published:** 2019-07-19

**Authors:** Yehee Hong, Sanghun Lee, Sungho Won

**Affiliations:** 1 Department of Public Health Sciences, Graduate School of Public Health, Seoul National University, Seoul, South Korea; 2 Department of Medical Consilience, Graduate School, Dankook University, Yongin, South Korea; 3 Interdisciplinary Program for Bioinformatics, College of Natural Science, Seoul National University, Seoul, South Korea; 4 Institute of Health and Environment, Seoul National University, Seoul, South Korea; King's College London, UNITED KINGDOM

## Abstract

Metformin, a first-line treatment for type 2 diabetes mellitus (T2DM), has recently been recognized for its pleotropic anti-proliferative, anti-cancer, and anti-aging effects. Contrary to the studies characterizing metformin effects in prostate cancer, little is known about these effects in BPH progression. With the Sample Cohort DB data during 2007 and 2017 from the Health Insurance Review and Assessment Service (HIRA) in South Korea, we investigated the preventative effect of metformin on BPH progression. The study population consisted of 211,648 BPH naïve patients that were diagnosed with BPH in 2009 and a follow-up occurrence of prostatectomy until 2017 that was defined as progression of BPH. These patients were divided into three treatment groups: without T2DM, T2DM without metformin, and T2DM with metformin. The hazard ratio in the T2DM with metformin group was 0.86 for prostatectomy compared to the group without T2DM (CI = 0.77–0.96, P value = 0.007) after adjusting for confounding factors such as age, comorbidity, residential area, level of hospital, and category of BPH medications. The T2DM with high-dose metformin group had a significantly lower risk of prostatectomy with hazard ratio of 0.76 (CI = 0.62–0.92, P value = 0.005) in stratified analysis. Our results suggest that metformin may improve BPH progression based on the reduced risk of prostatectomy, although T2DM effects on BPH were unclear. Future observational studies and prospective trials are needed to confirm the effects of metformin on BPH progression.

## Introduction

Benign prostate hyperplasia (BPH) is an age-related medical condition that is not generally regarded as a preventable ailment and is a high socioeconomic burden because it requires clinical intervention [1]. The pathogenesis of BPH is not clearly understood, but metabolic derangements including obesity, insulin resistance, and metabolic syndrome are known to have an exacerbating role in prostate enlargement [2, 3]. The prostate volume in BPH patients is significantly affected by type 2 diabetes mellitus (T2DM) in a prospective study, which suggested that it is positively correlated with obesity and fasting glucose level [4, 5]. In practice, men with T2DM are likely to have more severe BPH symptoms and lower urine flow rates compared to non-diabetic men, which is true of metabolic syndromes with underlying insulin resistance [4, 6]. However, the association between BPH and T2DM was not always observed [7, 8], while the consistent and strong evidence was provided that T2DM is significantly and inversely associated with prostate cancer [9, 10]. Therefore, it is necessary to investigate the role of T2DM treatment in BPH progression and prostate cancer.

Drugs for T2DM treatment including biguanide, sulfonylurea, meglitinide, thiazolidinedione, alpha glucosidase inhibitor, dipeptidyl peptidase inhibitors and sodium-glucose co-transporter 2 inhibitors have been developed and prescribed [11]. Metformin is an insulin-sensitizing oral biguanide that decreases insulin secretion, inhibits gluconeogenesis and energy consuming processes, and stimulates glycolysis and fatty acid oxidation; while sulfonylurea increases insulin release from the beta cells in the pancreas. Therefore, metformin promotes a shift from anabolic to catabolic metabolism and inhibits cell proliferation, which also suggests it could have an anti-cancer effect [12]. Epidemiological studies have shown that metformin users had a decreased risk of prostate cancer compared to non-users or those prescribed other oral antidiabetic medication [13, 14]. There are data on the beneficial effects of metformin on the recurrence, progression and survival of prostate cancer [15, 16]; however, large epidemiologic studies of metformin on BPH that adjust for sociodemographic factors and comorbid conditions are very limited despite the preclinical data that suggests metformin inhibits BPH progression by inhibiting insulin-like growth hormone 1 (IGF-1), which influences the proliferation of prostatic tissue and altered male hormone activity in the prostate [17, 18].

For these reasons, the present study aimed to examine the association between metformin and BPH progression using population-based medical evidence provided by the Health Insurance Review & Assessment Service (HIRA), which is a nation-wide and relatively accurate reporting service due to its coverage to people who reside in South Korea. As BPH progresses, pharmacological treatments such as alpha blockers and/or 5 alpha-reductase inhibitors (5ARI) are recommended, as well as various surgical interventions are suggested [19]. The occurrence of prostatectomy after diagnosis of BPH was considered a definite and objective outcome of clinical progression, compared to subjective patient-reported symptoms of BPH [20]. We hope that our study contributes to a better understanding of the effects of metformin on BPH progression.

## Subjects and methods

### Data source and study design

All data/samples were fully anonymized before we accessed them and our study was approved by the institutional review board of the Seoul National University (E1803/001-002). We used the Sample Cohort DB data during 2007 and 2017 from HIRA, a national database that included the diagnosis and treatment of BPH in all nationwide patients newly diagnosed with BPH in 2009 (index year). This cohort included men over 40 years old, which was based on the N40 disease code indicating BPH as described in the International Classification of Diseases 10th revision (ICD-10), as well as alpha blocker or 5ARI prescriptions that are listed in detail in supporting information (S1 Table). The prior history of the patients should not include diagnosis of BPH nor prescription of alpha blockers and 5ARI medications between 2007 and 2008. Patients who had a diagnosis of prostate cancer, inflammatory prostate diseases, neurological diseases, conditions that could affect lower urinary tract symptoms, and prostate surgical interventions were also excluded from the study (S2 Table). According to these criteria, the remaining subjects were defined as BPH naïve patients.

The BPH naïve population was classified into two groups according to their medical history of T2DM E11-E14 codes (ICD-10) between 2007 and 2008. The BPH naïve patients with T2DM were further classified into two subgroups depending on whether or not they were prescribed metformin (S1 Table). The occurrence of prostate surgical interventions such as transurethral resection, open prostatectomy, photoselective vaporization, and holmium laser enucleation in the follow-up period was defined as clinical BPH progression. The case of surgical interventions owing to newly development or subsequent diagnosis of new prostate cancer was not included.

To control for confounding factors, we included a set of baseline covariates that were potentially associated with BPH and the risk of BPH progression was defined as prostatectomy in the statistical models. The Charlson Comorbidity Index (CCI) is a method of weighting comorbidity conditions and was calculated for each patient using an ICD-10 algorithm [21]. Moreover, the initial BPH medications including alpha blockers and 5ARI were categorized into three groups: (1) alpha blocker only, (2) 5ARI only, and (3) combination with both drug treatments. In addition, age, residential area, and level of hospital at BPH diagnosis were also considered in the study.

This study was approved by the institutional review board of the Seoul National University (E1803/001-002).

### Statistical analysis

Demographic and clinical characteristics of subjects are reported as mean ± standard deviation for continuous variables, and as frequencies and percentages for categorical variables. Continuous variables were analyzed by independent sample t-tests and categorical variables were analyzed using the chi-squared test. We calculated the hazard ratio (HR) for comparisons between the BPH without T2DM group and two BPH with T2DM subgroups with and without metformin treatment. Patients were censored if they reached the end of the study period without any prostatectomy. Cox proportional hazard models were adjusted for potential confounding factors before they were used to analyze the effect of T2DM or metformin on BPH progression, which were expressed as HR and 95% confidence intervals (CI). The proportional hazard assumption was also supported by the survival curves for the two strata, BPH with T2DM and BPH without T2DM, having hazard functions that were proportional over time, constant relative hazard (P value = 0.357). In the stratified analysis, the total daily dosage of metformin was calculated for each patient during the prior two-year period (2005–2006) to consider potential dosage effects of metformin on prostatectomy. After cumulative dosage was calculated, the T2DM with metformin group was sub-categorized into low, middle, and high metformin dosage groups. P values of < 0.05 were considered statistically significant.

## Results

**Fig 1:**
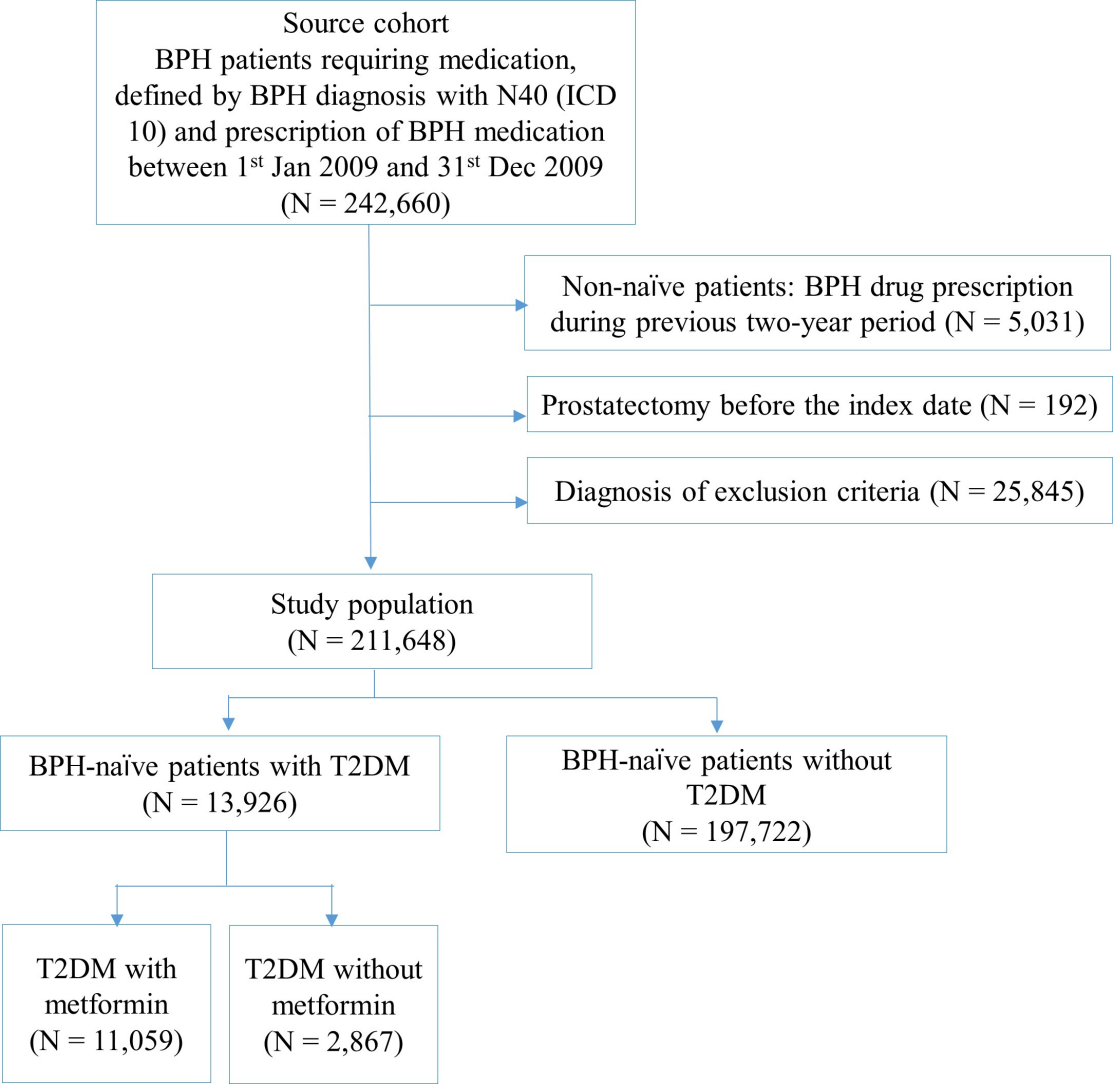
The flowchart for the study design.

A total of 211,648 patients were included in the study population after inclusion and exclusion criteria was met. These patients were sorted into three groups: (1) T2DM+metformin (N = 11,059), (2) T2DM-metformin (N = 2,867), (3) no T2DM (N = 197,722), which is shown in Fig 1. Based on baseline characteristics, patients’ age was significantly different between groups and the distribution of age in each group was also shown by age groups like 40s, 50s, 60s, and over 70s in Table 1. Residential area and CCI were significantly different between groups depending on their T2DM status, and residential area was significantly different between groups according to metformin prescription (Table 1).

**Table 1:** Baseline characteristics according to T2DM and metformin medication comorbidity.

	Total	BPH without T2DM	BPH with T2DM	P-value	T2DM without metformin	T2DM with metformin	P-value
	N (%)	N (%)	N (%)		N (%)	N (%)	
Age	61.4 ± 11.0	61.2 ± 11.0	64.9 ± 9.4	<0.0001	65.9 ± 9.6	64.7 ± 9.4	<0.0001
40	33,523 (15.8)	32,681 (16.5)	842 (6.0)		151 (5.3)	691 (6.2)	
50	60,136 (28.4)	57,036 (28.9)	3100 (22.3)		582 (20.3)	2,518 (22.8)	
60	64,933 (30.7)	59,601 (30.1)	5332 (38.3)		1,076 (37.5)	4,256 (38.5)	
> = 70	53,056 (25.1)	48,404 (24.5)	4652 (33.4)		1,058 (36.9)	3,594 (32.5)	
Residential area				<0.0001			<0.001
Seoul (Capital)	43,399 (20.5)	40,794 (20.6)	2,605 (18.7)		580 (20.2)	2,025 (18.3)	
Metropolitans	60,239 (28.5)	56,284 (28.5)	3,955 (28.4)		842 (29.4)	3,113 (28.2)	
Small town	108,010 (51.0)	100,644 (50.9)	7,366 (52.9)		1,445 (50.4)	5,921 (53.5)	
BPH medication				0.469			0.596
Alpha blocker	143,815 (67.9)	134,402 (67.9)	9,413 (67.6)		1923 (67.0)	7489 (67.8)	
5ARI only	15,409 (7.3)	14,361 (7.3)	1,048 (7.5)		227 (7.9)	820 (7.4)	
Combination	52,424 (24.8)	48,959 (24.8)	3,465 (24.9)		722 (25.1)	2741 (24.8)	
CCI				0.038			0.996
≤1	167,479 (79.1)	156,498 (79.1)	10,981 (78.9)		8,734 (79.0)	2,247 (78.4)	
2–3	26,214 (12.4)	24,463 (12.4)	1,751 (12.6)		1,394 (12.6)	357 (12.5)	
≥4	17,927 (8.5)	16,738 (8.5)	1,189 (8.5)		927 (8.4)	262 (9.1)	

The Cox proportional hazard models were adjusted with confounding factors such as CCI, age, residential area, level of hospital, and category of BPH medications. With these adjustments, the models showed that the HR in the T2DM with metformin group was 0.86 for prostatectomy compared to the group without T2DM (CI = 0.77–0.96, P value = 0.007; Table 2). Therefore, BPH-naïve patients treated with metformin had a significantly lower risk of prostatectomy. The adjusted covariates were age, the urban area related to economic status, BPH medication combination associated with BPH severity, and the high level of hospital at diagnosis representing the convenience of prostatectomy. In the assessment of the data with respect to the effect of these covariates indicated significantly high HR of prostatectomy in all groups (Table 2). Based on the total daily dose of metformin, stratified analysis showed that the T2DM group that received high-dose metformin treatment had a significantly lower risk of prostatectomy with an HR of 0.76 (CI = 0.62–0.92, P value = 0.005; Table 3).

**Table 2:** Hazard ratios for prostatectomy according to T2DM, metformin, and baseline characteristics.

	HR^1^	95% CI		P-value
T2DM				
No (reference)				
Yes without metformin	0.94	0.78	1.14	0.557
Yes with metformin	0.86	0.77	0.96	0.007
Age	1.05	1.04	1.05	<0.0001
CCI	1.00	0.99	1.02	0.587
Residential area				
Capital (reference)				
Metropolitan areas	0.80	0.75	0.85	<0.0001
Small town	0.69	0.66	0.73	<0.0001
BPH medication				
Alpha blocker only (reference)				
5ARI only	0.99	0.90	1.09	0.811
Combination	1.40	1.34	1.48	<0.0001
Level of hospital at diagnosis				
Primary (reference)				
Secondary	1.27	1.19	1.32	<0.0001
Tertiary	1.32	1.57	1.51	<0.0001

^1^Hazard ratio calculated according to Cox proportional hazards regression after adjustments for age, area of hospital, initial BPH medication, hypertension, and outpatient department visits.

**Table 3:** Hazard ratios for prostatectomy according to T2DM and total daily dose of metformin.

	HR	95% CI		P-value
BPH without T2DM (Reference)				
T2DM without metformin	0.94	0.78	1.14	0.556
T2DM with metformin				
Low metformin dosage	0.88	0.73	1.06	0.165
Middle metformin dosage	0.95	0.79	1.13	0.526
High metformin dosage	0.76	0.62	0.92	0.005

Soon after the index date of BPH diagnosis, prostatectomy might be influenced by other factors besides metformin. To verify consistency of our results, sensitivity analysis of HR for prostatectomy was carried out after excluding prostatectomy that occurred one to four years after the index date. In this analysis, patients in the T2DM with metformin groups showed consistently and significantly lower HRs compared to the patients without T2DM at any washout periods up to four years (S3 Table).

## Discussion

Patients with obesity, metabolic syndrome, and T2DM have risk for BPH, which is explained by the associated insulin resistance and hyperglycemia pathophysiological mechanisms as followed [22]. Insulin influences sympathetic nervous system activity, which contributes to more severe lower urinary tract symptoms as well as increases the transcription of genes involved in sex hormone metabolism and thereby influences prostate stromal and epithelial cell growth. Hyperglycemia not only increases the production of sex hormones, but also influences the viability of parasympathetic neurons. Vascular damage induced by T2DM can also lead to hypoxia, which is proposed to contribute to the pathogenesis of BPH in T2DM patients [23]. Due to these overlapping mechanisms of neuropathic, hormonal, and microvascular dysfunctions, previous studies have reported an association between T2DM and prostate disease but failed to differentiate the dynamic components of lower urinary tract function from BPH progression [7, 24]. Moreover, various confounding factors such as sleep apnea and medications like diuretics for hypertension may lead to lower urinary tract symptoms [25]. A large population‐based study in an Asian population showed that the significant association between T2DM and BPH was confounded with factors such as hypertension, coronary heart disease, and hyperlipidemia [26]. Among these confounding factors, the clinical studies that included medication adjustments for T2DM were also limited, but metformin in T2DM could affect the prognosis of BPH based on solid evidence from preclinical data [17, 18].

This seven-year follow-up observational study demonstrates that the incidence of prostatectomy in BPH-naïve patients categorized by T2DM comorbidity significantly decreased with metformin administration. The multivariate analysis adjusted for the confounding factors of age, disease comorbidity, economic status, BPH severity, and prostatectomy availability presented that metformin had a beneficial effect on BPH progression. Although our study did not evaluate BPH symptoms such as acute urinary retention, urinary incontinence, and renal insufficiency, prostatectomy as the endpoint could be more objectively represented by BPH progression. The BPH related surgery had been included as a definition for BPH clinical progression in well-designed clinical trials for medical drugs related to BPH [27, 28].

Metformin has become one of the most widely used first-line medications for the treatment of T2DM and is generally well tolerated, with long history of used for over 60 years [12]. Insulin sensitivity is improved by metformin, which increases peripheral glucose uptake and utilization as well as decreases hepatic glucose production and intestinal glucose absorption; while symptoms like sulfonylureas, hyperinsulinemia or weight gain are not caused. In addition to lowered glucose, pleotropic effects of metformin on various diseases have been observed, for example antitumor, antiaging, cardiovascular protective, and neuroprotective effects; metformin could also be an optional treatment for polycystic ovary syndrome [29]. In particular, the inhibitory effect of metformin on BPH is due to the down-regulation of the IGF-1 signaling pathway that shares similar biosynthetic sequences with insulin, which is key in prostatic epithelial cell growth, and regulating the stromal-epithelial interaction through the paracrine pathway between IGF-1 and IGF-1 receptor in the prostate [18, 30]. Metformin also blocks the cell cycle in the G0/G1 phase through the inhibition of cyclin protein expression under the IGF-1 axis, specifically cyclin D [18, 31]. Additionally, the expression of estrogen receptor-β through induction of a pro-apoptotic cascade was upregulated with metformin treatment, while estrogen receptor-α, proliferative and anti-apoptotic responses was down-regulated [17, 32]. To our best knowledge, our study provides initial evidence for the plausible effect of metformin on BPH in population-based cohort data. High doses of metformin in our data were significantly associated with a reduction in prostatectomy compared to middle or low dose treatments. Furthermore, sensitivity analysis also confirmed that metformin is beneficial to BPH patients after washout periods up to four years.

There are some limitations to the present study. The database did not include clinical variables such as prostate volume and blood tests. First, the number of T2DM patients with BPH might have been underestimated because only patients based on BPH and T2DM diagnosis codes in hospitals were included in the analysis. Second, the discrepancy regarding the actual amount of metformin intake exists, because this study was done with the national data with the claimed prescription, not the collected prescription. Third, the incidence of surgery might have been underestimated as well, since patients diagnosed with acute urinary retention were excluded. Finally, confounding factors, like statins, antihypertensive drugs or anti-inflammatory drugs was not taken into account in spite of CCI consideration for comorbidity. The dosage association of metformin might occur the same proportion as other drugs due to increasing severity of other diseases or T2DM related causes. Therefore, more drug variables should be included in the future study to validate our findings. Despite these limitations, our data support the hypothesis that metformin use improves the BPH outcome in T2DM patients, although T2DM itself may increase the risk of BPH. It also encourages well-conducted observational studies and prospective trials to confirm the effects of metformin on BPH progression.

## Supporting information

S1 Table Medication for BPH and T2DM. (DOCX)

S2 Table Exclusion criteria of diseases and prostatectomy. (DOCX)

S3 Table Sensitivity analysis of hazard ratios for prostatectomy according to T2DM and metformin after excluding prostatectomy occurring from 1 to 4 years after the index date. (DOCX)
