# Supplementary material for: The preventive effect of metformin on progression of benign prostate hyperplasia: A nationwide population-based cohort study in Korea
Source: PLoS One. 2019 Jul 19;14(7):e0219394. doi: 10.1371/journal.pone.0219394 (PMC6641083; doi:10.1371/journal.pone.0219394)
Supplement: S1 Table — (DOCX) [file pone.0219394.s001.docx]

**S1 Table.** Medication for BPH and T2DM

| Category | Molecule | Code |
| --- | --- | --- |
| BPH medications |  |  |
| Alpha blocker | Tamsulosin | 234601AXX, 234603ACR |
|  | Alfuzosin | 104803ATR |
|  | Doxazosin | 14910XXXX |
|  | Terazosin | 23550XXXX |
|  | Silodosin | 50420XXXX |
|  | Naftopidil | 61420XXXX |
| 5ARI (5-alpha-reductase-inhibitor) | Dutasteride | 458801ACS |
|  | Finasteride | 159001ATB |
| T2DM medications |  |  |
| Biguanide | Metformin | 19150XXX |
| Sulfonylurea | Glibenclamide | 165402ATB |
|  | Gliclazide | 16560XXXX |
|  | Glimepiride | 16570XXXX |
|  | Glipizide | 165801ATB |
|  | Gliquidone | 165901ATB |
| Meglitinide | Mitiglinide | 486101ATB |
|  | Nateglinide | 43020XXXX |
|  | Repaglinide | 37950XXXX |
| Thiazolidinedione | Pioglitazone | 43190XXXX |
|  | Rosiglitazone | 34800XXXX |
| Alpha glucosidase inhibitor | Acarbose | 10060XXXX |
|  | Voglibose | 24900XXXX |
| Metformin combination | Glibenclamide | 471900ATB, 421100AT, 443400ATB, 443500ATB |
|  | Glimepiride | 474200ATB, 474300ATB |
|  | Rosiglitazone | 452900ATB, 452700ATB, 469100ATB, 471800ATB |
