# Supplementary material for: The preventive effect of metformin on progression of benign prostate hyperplasia: A nationwide population-based cohort study in Korea
Source: PLoS One. 2019 Jul 19;14(7):e0219394. doi: 10.1371/journal.pone.0219394 (PMC6641083; doi:10.1371/journal.pone.0219394)
Supplement: S2 Table — (DOCX) [file pone.0219394.s002.docx]

**S2 Table.** Exclusion criteria of diseases and prostatectomy

| DIAGNOSIS | | ICD-10 codes |
| --- | --- | --- |
| Malignant neoplasm of the prostate | | C61 |
| Parkinson’s disease | | G20 |
| Secondary parkinsonism | | G21 |
| Parkinsonism in disease classified elsewhere | | G22 |
| Dementia in Parkinson’s disease (G20+) | | F02.3 |
| Multiple sclerosis | | G35 |
| Hemiplegia, cerebral palsy with other paralytic syndromes, other paralytic syndromes | | G80-G83 |
| Cerebrovascular diseases | | I60-I69 |
| Neoplasm of uncertain origin or unknown behavior of the prostate | | D40.0 |
| Benign neoplasm of the prostate | | D29.1 |
| Acute urinary retention | | R33 |
| Inflammatory diseases of the prostate | | N41 |
| Thermal therapy | | R3516 |
| Prostatectomy | Transurethral resection | R3975 |
|  | Open prostatectomy | R3950 |
|  | Photoselective vaporization | R3976 |
|  | Holmium laser enucleation | R3977 |
