# Supplementary material for: The preventive effect of metformin on progression of benign prostate hyperplasia: A nationwide population-based cohort study in Korea
Source: PLoS One. 2019 Jul 19;14(7):e0219394. doi: 10.1371/journal.pone.0219394 (PMC6641083; doi:10.1371/journal.pone.0219394)
Supplement: S3 Table — (DOCX) [file pone.0219394.s003.docx]

**S3 Table.** Sensitivity analysis of hazard ratios for prostatectomy according to T2DM and metformin after excluding prostatectomy occurring from 1 to 4 years after the index date.

|  | No T2DM | T2DM without metformin | T2DM with metformin |
| --- | --- | --- | --- |
| Washout of 1 year |  |  |  |
| HR (95% CI) | 1.00 (reference) | 0.94 (0.75-1.19) | 0.81(0.71-0.93) |
| P-value |  | 0.610 | 0.002 |
| Washout of 2 years |  |  |  |
| HR (95% CI) | 1.00 (reference) | 1.00(0.78-1.29) | 0.83(0.72-0.96) |
| P-value |  | 0.996 | 0.014 |
| Washout of 3 years |  |  |  |
| HR (95% CI) | 1.00 (reference) | 0.98(0.74-1.30) | 0.83(0.70-0.98) |
| P-value |  | 0.883 | 0.024 |
| Washout of 4 years |  |  |  |
| HR (95% CI) | 1.00 (reference) | 1.03(0.75-1.41) | 0.79(0.65-0.96) |
| P-value |  | 0.870 | 0.016 |
